# Phase II Cardiac Rehabilitation Under Compulsory Insurance in Kazakhstan: A Five-Year Cohort Analysis of Clinical and Economic Outcomes

**DOI:** 10.3390/jcm14176317

**Published:** 2025-09-07

**Authors:** Yelena Sergeyeva, Lyudmila S. Yermukhanova, Ardak N. Nurbakyt, Gulnara L. Kurmanalina, Dariush Walkowiak, Maral G. Nogayeva, Alireza Afshar

**Affiliations:** 1 Department of Public Health and Public Health Care, West-Kazakhstan Marat Ospanov Medical University, 030019 Aktobe, Kazakhstan; y.sergeyeva2025@gmail.com; 2 Department of Public Health, Asfendiyarov Kazakh National Medical University, 050012 Almaty, Kazakhstan; a.nurbakyt@kaznmu.kz; 3 Department of Internal Medicine No. 2, West-Kazakhstan Marat Ospanov Medical University, 030019 Aktobe, Kazakhstan; gl.kurmanalina@gmail.com; 4 Department of Organization and Management in Health Care, Poznan University of Medical Sciences, 61-701 Poznan, Poland; dariuszwalkowiak@ump.edu.pl; 5 Department of Rheumatology, Asfendiyarov Kazakh National Medical University, 050067 Almaty, Kazakhstan

**Keywords:** cardiac rehabilitation, acute myocardial infarction, left ventricular ejection fraction, health economics, Kazakhstan

## Abstract

**Background/Aim of Study:** Cardiovascular diseases (CVDs) are the leading cause of morbidity and mortality globally. Cardiac rehabilitation (CR) plays a pivotal role in the recovery of post-acute myocardial infarction (AMI) patients. Despite evidence supporting its clinical benefits, CR remains underutilized, especially in middle-income countries like Kazakhstan. This study aimed to evaluate the clinical effectiveness and economic impact of phase II CR among patients with AMI treated at the Almaty City Cardiology Center between 2018 and 2022. **Methods**: A retrospective cohort study was conducted using data from 2672 AMI patients. Two cohorts were compared: those who participated in phase II CR and those who did not. Primary outcomes included changes in left ventricular ejection fraction (LVEF), rehospitalization rates, and return to active work. **Results**: Economic outcomes involved direct medical costs related to initial hospitalization and follow-up care. CR participants showed significant improvements in LVEF (53.7% vs. 49.0% in non-CR patients, *p* < 0.001). Despite these clinical benefits, there was no significant reduction in long-term treatment costs between the CR and non-CR groups. CR users had slightly higher initial treatment costs but similar cumulative costs for subsequent treatments over two years. Importantly, government funding limitations were found to hinder the full effectiveness of CR programs in Kazakhstan. **Conclusions**: Phase II CR improves cardiac function in AMI patients but does not reduce long-term treatment costs. The current insufficient government funding for CR limits its broader impact. Expanding CR services and increasing funding are essential to maximize its benefits within Kazakhstan’s healthcare system.

## 1. Introduction

Cardiovascular diseases (CVDs) remain the leading cause of morbidity and mortality worldwide, responsible for an estimated 17.9 million deaths annually, equivalent to 32% of all global deaths [1]. Acute myocardial infarction (AMI), a major manifestation of ischemic heart disease, contributes substantially to this burden, with survivors at high risk for recurrent events, heart failure, and impaired quality of life [2]. In response, exercise-based cardiac rehabilitation (CR) has been established as a cornerstone of secondary prevention, demonstrating reductions in cardiovascular mortality by approximately 26% and hospital readmissions by 18% across diverse patient populations [3,4].

Despite robust evidence supporting its clinical efficacy, CR remains underutilized, with global participation rates seldom exceeding 30% even in high-income countries [5]. Cost-effectiveness analyses conducted predominantly in Western European and North American settings have indicated that CR is highly cost-effective, often yielding incremental cost-effectiveness ratios well below commonly accepted willingness-to-pay thresholds [6,7,8]. Yet extrapolation of these findings to low- and middle-income countries is problematic given differences in healthcare financing, baseline event rates, and unit costs [7].

In Central Asia, and Kazakhstan in particular, systematic data on CR infrastructure, utilization, and economic impact are sparse. National health reports document a rising incidence of AMI over the past decade, with centralized cardiac care centers increasingly equipped to deliver advanced interventional therapies [9]. However, formalized CR programs at the secondary (outpatient or “phase II”) level were only recently integrated into the framework of Kazakhstan’s compulsory medical insurance, and their real-world performance remains largely uncharacterized [10]. Anecdotal accounts suggest delays in program initiation, heterogeneity in session content, and variable patient adherence, underscoring the need for rigorous evaluation.

Moreover, the interplay between CR participation and long-term economic outcomes within Kazakhstan’s healthcare system is unknown. While CR may impose modest upfront costs related to staff, facilities, and exercise equipment, these may be offset by reductions in recurrent hospitalizations, emergency admissions, and long-term disability [11,12]. A comprehensive assessment of direct medical expenditures across index hospitalization, CR delivery, and follow-up care is therefore critical to inform policy decisions regarding resource allocation and potential scale-up of CR services under the compulsory insurance mechanism.

Against this backdrop, the present study aims to evaluate both the clinical effectiveness and the economic impact of phase II CR among patients with AMI treated at the Almaty City Cardiology Center between 2018 and 2022. Specifically, we compare functional recovery measured by improvements in left ventricular ejection fraction (LVEF), rehospitalization rates, and return to active professional life between CR participants and non-participants and conduct a detailed cost analysis from the payer’s perspective.

### Aim of Study

By integrating retrospective clinical and financial data, we seek to generate locally relevant evidence to guide optimization of CR delivery within Kazakhstan’s evolving healthcare infrastructure. Specifically, this study aims to evaluate the clinical outcomes, disability rates, and long-term treatment costs associated with phase II cardiac rehabilitation in patients with acute myocardial infarction, thereby informing policy decisions on its effectiveness, efficiency, and potential for broader implementation.

## 2. Materials and Methods

### 2.1. Study Design and Setting

We conducted a retrospective cohort study of medical records from patients diagnosed with AMI who received care at the Almaty City Cardiology Center, a tertiary referral institution providing comprehensive cardiovascular services under Kazakhstan’s compulsory medical insurance system. Data were extracted for admissions occurring between 1 January 2018 and 31 December 2022. In the context of Kazakhstan’s healthcare system, the phase II CR program evaluated in this study was implemented under conditions of limited program funding, scope, and intensity. The intervention consisted of a structured outpatient program delivered at the Almaty City Cardiology Center, with a duration ranging from approximately 6 to 9 weeks (mean ± SD: 1.89 ± 0.49 months) [13]. This duration reflects the total time from enrollment to completion of the course, with sessions typically held two to three times per week rather than continuous inpatient care. The program adhered to core international CR components, including supervised exercise sessions, patient education, and medical optimization, but its scale and resources reflected the constraints of the national compulsory medical insurance system. These contextual factors should be considered when interpreting both the clinical and economic outcomes of the program.

Post-Phase II Follow-up: After completing the phase II cardiac rehabilitation program, patients were followed in outpatient cardiology and primary care clinics, with routine assessments and reinforcement of lifestyle recommendations. Standard pharmacotherapy included dual antiplatelet therapy, beta blockers, angiotensin-converting enzyme inhibitors or angiotensin receptor blockers, and statins; mineralocorticoid receptor antagonists were added when indicated. Most essential medications were reimbursed through the national compulsory medical insurance scheme, with limited co-payment for certain newer agents.

### 2.2. Study Population and Group Formation

**Figure 1:**
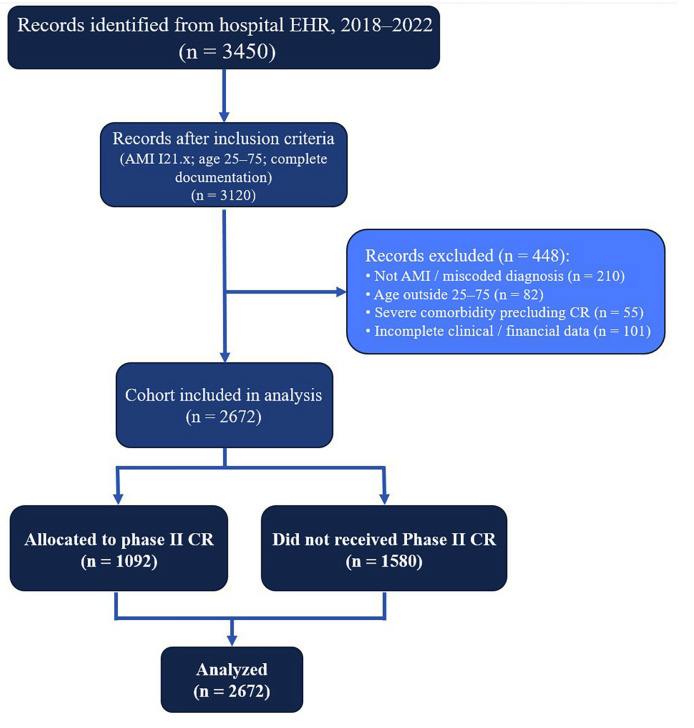
Flow diagram illustrating patient selection, inclusion, and allocation in the retrospective cohort study.

Eligible records were those of patients aged 25–75 years, of both sexes, with a primary discharge diagnosis of AMI (ICD-10 codes I21.x) and complete documentation of index hospitalization, treatment strategy, and follow-up care (Figure 1). Exclusion criteria comprised pregnancy, congenital heart disease, severe musculoskeletal or psychosomatic comorbidities precluding participation in CR, and incomplete financial and medical data. From the eligible pool, we formed two cohorts:

- CR group: Patients who completed second-stage (phase II) cardiac rehabilitation, which, in Kazakhstan, is delivered as an inpatient program in specialized hospital departments. In accordance with local health policy and similar to other post-Soviet countries, completion is determined by fulfilling an individualized number of prescribed bed days according to a standardized national protocol rather than a fixed number of exercise sessions [10,14,15]. This approach reflects both the regulatory framework and clinical practice in the region.
- Non-CR group: Patients who received the same standard therapy, but without a course of rehabilitation treatment (without the 2nd stage of rehabilitation).

Group allocation also accounted for index-treatment-modality surgical coronary artery bypass grafting (CABG), percutaneous coronary intervention (PCI), or conservative pharmacological management to control for baseline intervention differences.

### 2.3. Sample Size

A total of 2672 patient records met the inclusion criteria, comprising 1092 in the CR group and 1580 in the non-CR group. This sample afforded >80% power to detect a between-group difference of 5% in mean LVEF improvement at α = 0.05, based on prior data estimating a standard deviation of 12% [3].

### 2.4. Data Collection

Demographic variables (age, sex, residence), clinical data (AMI type, LVEF at admission, and follow-up echocardiography), and treatment details (CABG, PCI, or medical therapy) were abstracted from the electronic health record. Economic data included direct medical costs of the index hospitalization, CR sessions (personnel, facility overhead, and consumables), and any cardiovascular (acute conditions)-related rehospitalizations over a two-year follow-up. Costs were captured in KZT and converted to USD using the annual average exchange rate for each calendar year.

### 2.5. Primary Outcomes

The primary clinical outcome was changes in LVEF between baseline (within 2 weeks post-AMI) and follow-up (at 6–12 months). LVEF was assessed by transthoracic echocardiography using the same model of ultrasound machine for all participants. All examinations were performed according to a standardized protocol, and LVEF was calculated using Simpson’s biplane method. To minimize intra- and inter-observer variability, a single experienced cardiologist reviewed all measurements, and duplicate assessments were performed on a random 10% subsample, yielding an intra-observer coefficient of variation below 5%. Secondary outcomes included frequency of cardiovascular rehospitalizations (AMI, unstable angina), total bed days, and rate of return to active professional life or disability status at 24 months. The economic outcome was cumulative direct medical cost per patient over the index admission plus a two-year follow-up period.

### 2.6. Statistical Analysis

Continuous variables are presented as means ± standard deviation or medians (interquartile range) based on distribution. Categorical variables are reported as counts and percentages. Between-group comparisons utilized Student’s t-test or the Mann–Whitney U test for continuous data and the χ^2^ or Fisher’s exact test for categorical data. A univariate general linear model assessed the independent effect of CR participation on follow-up LVEF, adjusting for baseline LVEF and treatment modality. All tests were two-sided, with *p* < 0.05 denoting statistical significance. Analyses were performed using SPSS v.26 (IBM Corp., Armonk, NY, USA).

### 2.7. Ethical Considerations

The Institutional Review Board of the West-Kazakhstan Marat Ospanov Medical University approved the study protocol (Approval No. ACCC-2023-042) and waived individual consent given the retrospective design and de-identified data abstraction.

## 3. Results

### 3.1. Socio-Demographic and Clinical Characteristics

The number of the participants in this study was 2672 of the Kazakh population. Table 1 compares age, sex, education, residence, initial MI types, comorbid disabilities, and treatment modalities at first hospitalization between CR users and non-users. As shown in this table, the mean age of participants was 53.8 years (±6.6), with no significant difference between CR users (53.87 years) and non-users (53.79 years; *p* = 0.766). The sample was overwhelmingly male (90.2%), with a non-significant trend toward slightly more men among non-users (91.1% vs. 89.0%; χ^2^(1) = 3.124, *p* = 0.077). Educational attainment, categorized from incomplete secondary to university level, did not differ by rehabilitation status (χ^2^(4) = 1.298, *p* = 0.862). Urban residency predominated (81.1% overall; 82.7% CR users vs. 80.0% non-users; χ^2^(1) = 3.054, *p* = 0.081), and the vital status at two years was similar between groups (currently alive: 87.9% vs. deceased: 12.1%; *p* = 0.091).

Notably, current disability was more frequent among CR users (14.4% vs. 10.9%; χ^2^(1) = 7.290, *p* = 0.007), and CR users were more likely to remain on disability over the two-year observation (43.1% vs. 37.3%; χ^2^(1) = 9.244, *p* = 0.002), with a longer mean duration of disability (9.68 months vs. 8.21 months; *p* = 0.001). There were no differences in primary versus recurrent MI at first hospitalization (*p* = 0.747), but CR users had a slightly higher proportion of STEMI (58.5% vs. 54.5%; χ^2^(1) = 4.244, *p* = 0.039). The treatment type for index MI also differed: CR users were less likely to receive drug-only therapy (21.3% vs. 24.2%) and more likely to undergo CABG (18.3% vs. 14.8%; χ^2^(2) = 7.194, *p* = 0.027).

### 3.2. Initial Hospitalization Costs and Rehabilitation Timing

The study population comprised both patients who participated in cardiac rehabilitation (CR users) and those who did not (non-CR users), with CR uptakes at first hospitalization of 40.9% versus 59.1% (Table 2). This table presents data on the proportion of CR participants, associated index treatment costs, and average time taken to complete phase II rehabilitation. The results show that the mean direct treatment cost at index MI was USD 1988.80 among CR users versus USD 1879.36 in non-users (*p* = 0.019). In local currency, this corresponded to 1,046,736.86 KZT versus 989,139.52 KZT. The average period of rehabilitation to complete the cardiac rehabilitation course was 1.89 months among those who attended.

### 3.3. Change in Left Ventricular Ejection Fraction

Table 3 summarizes the baseline and follow-up LVEF values and results from the univariate general linear model assessing the independent contribution of CR. The baseline LVEF was significantly higher in CR users (mean: 51.76%) compared with non-users (47.77%; *p* < 0.001). At follow-up, the LVEF improved to 53.72% in the CR group versus 48.98% in non-users (*p* < 0.001). A univariate general linear model adjusted for baseline LVEF demonstrated that participation in rehabilitation was independently associated with greater second LVEF (Type III SS = 4173.99; F(1, 2669) = 74.02; *p* < 0.001; partial η^2^ = 0.027), even after accounting for the strong effect of initial LVEF (Type III SS = 117,133.40; F(1, 2669) = 2077.16; *p* < 0.001; partial η^2^ = 0.438). The model explained 46.7% of the variance in follow-up LVEF (R^2^ = 0.467).

Univariate general linear model:

- Dependent Variable: second LVEF
- Fixed Factor: rehabilitation (group: yes/no)
- Covariate: initial LVEF

### 3.4. Subsequent Hospitalizations, Length of Stay, and Costs

Table 4 details the rates of recurrent MI and unstable angina, hospitalization days, and associated treatment costs, stratified by CR participation. Over the first year post-MI, the proportion experiencing a repeat MI did not differ by CR status (yes: 43.7% CR vs. 56.3% non-CR; *p* = 0.515). The mean bed days (9.44 vs. 9.33; *p* = 0.865) and treatment costs for repeat MI (USD 3175.54 vs. 2955.51; *p* = 0.385) were similar. The emergency admissions for unstable angina in year 1 also showed no significant differences in frequency (42.2% vs. 57.8%; *p* = 0.711), length of stay, or costs.

During year 2 post-MI, repeat MI rates remained comparable (39.7% vs. 60.3%; *p* = 0.786), but CR users incurred significantly higher mean costs for repeat MI treatment (USD 2169.45 vs. 1745.67; *p* = 0.017; 1,141,817.16 KZT vs. 918,775.78 KZT; *p* = 0.017). There were no significant differences in two-year emergency admissions for unstable angina with respect to rates, bed days, or costs.

### 3.5. Cumulative Cost Comparison

**Figure 2:**
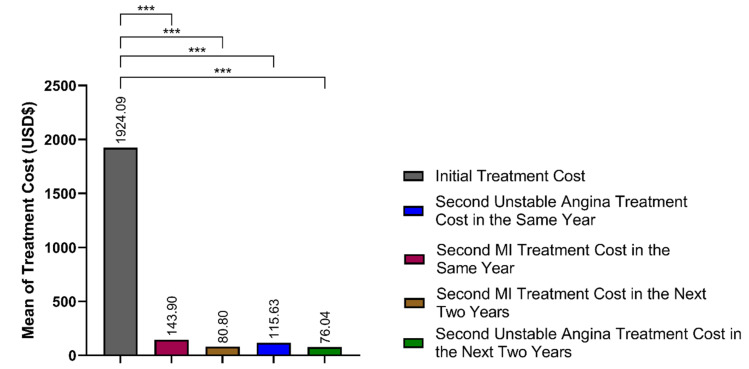
Cumulative treatment costs following acute myocardial infarction by cardiac rehabilitation status: a two-year economic comparison. This figure illustrates the initial hospitalization costs alongside subsequent expenditures for recurrent myocardial infarction and unstable angina over a 24-month period, highlighting the cost convergence between CR and non-CR groups. *** *p* < 0.001 for comparison between CR and non-CR groups.

Figure 2 illustrates that although CR users incurred higher upfront costs during index hospitalization (Table 2), their cumulative costs for subsequent MI and unstable angina treatments over the follow-up period converged with those of non-users. By combining initial and follow-up treatment expenses, CR participation appears cost-neutral over two years despite higher utilization of interventional procedures and slightly elevated repeat MI treatment costs in later follow-up.

## 4. Discussion

One of the most notable findings of this study is the statistically significant improvement in mean LVEF among CR users compared with non-users. The baseline LVEF was already marginally higher in CR users, which may reflect a referral bias, but the follow-up values indicate a persistent benefit beyond the initial differences. Even after adjusting for baseline LVEF using a univariate general linear model, CR participation remained an independent predictor of improved follow-up EF, consistent with previous randomized trials and meta-analyses demonstrating that exercise-based CR improves cardiac remodeling and systolic function [3,16].

Our results align closely with the findings of Anderson et al., who, in a Cochrane meta-analysis of 63 randomized trials, found that CR significantly improves functional capacity and modestly enhances LVEF, particularly in post-MI populations [3]. Moreover, Dibben et al. reported that aerobic exercise, a key component of phase II CR, leads to improvements in cardiac output and peripheral oxygen extraction, thus enhancing overall myocardial efficiency [4,17].

Importantly, this study contributes localized evidence to the growing global consensus that CR should be considered a standard component of AMI recovery. While the benefits of LVEF may appear modest (approximately 2%), even small gains in systolic function have been shown to correlate with reduced heart failure incidence and improved quality of life [18,19].

Contrary to expectations, we observed no statistically significant difference in rehospitalization rates between CR and non-CR participants for either repeat MI or emergency admissions for unstable angina across the two-year follow-up period. This finding diverges from several international studies where CR has consistently been associated with reduced rehospitalization and all-cause mortality [17,20]. For example, a nationwide U.S. cohort study by Suaya et al. demonstrated a 26% reduction in hospital readmission at one year in Medicare patients who completed CR after AMI [21].

Several factors could explain this apparent discrepancy. First, the nature of healthcare delivery in Kazakhstan, including access to primary care, patient self-management practices, and medication adherence, may moderate the protective effect of CR. Second, the baseline event rates for repeat MI and unstable angina were relatively low across both groups, limiting the statistical power to detect small differences. Third, the fidelity and intensity of CR delivery may vary significantly, and our dataset did not quantify actual CR session attendance or exercise volume, which are key determinants of outcomes [22,23].

Nevertheless, it is important to note that the similarity in readmission rates occurred despite higher comorbidity burdens among CR users, particularly higher baseline disability rates (14.4% vs. 10.9%) and slightly more STEMI cases (58.5% vs. 54.5%), suggesting that CR may have played a mitigating role in stabilizing these higher-risk individuals. Moreover, the CR group underwent more interventional procedures (e.g., CABG), which may have increased subsequent healthcare encounters for surveillance and follow-up, partially masking any readmission reduction effects.

The economic findings of this study are particularly relevant for policymakers. While CR users incurred slightly higher costs during initial hospitalization, the cumulative costs over the two-year period were similar between groups, despite the CR cohort receiving more advanced interventions and follow-up care. In fact, the treatment costs for repeat MI in the second year were significantly higher in the CR group, yet this did not translate to a significant difference in overall expenditures when viewed holistically across all cardiovascular-related admissions.

These findings suggest that cardiac rehabilitation in the context of Kazakhstan’s compulsory medical insurance system may not be sufficiently effective, primarily due to the limited financial resources allocated for its implementation. While rehabilitation programs offer potential benefits in theory, the current allocation of government funds fails to adequately support the scaling of CR services to achieve meaningful clinical improvements for patients.

Economic evaluations of CR programs have generally shown that these interventions are cost-effective in high-income countries [24,25]. However, when considering the context of Kazakhstan, the financial constraints surrounding CR delivery, such as inadequate government funding, undermine the potential for meaningful cost savings or long-term benefits. In countries like Brazil and Chile, CR has been found to be cost-saving due to reductions in hospital readmissions and long-term disability [26,27], but such outcomes are not fully realized in Kazakhstan, where limited resources restrict access to essential components of CR, including adequate staffing and comprehensive rehabilitation services [28].

While the results of this study show some improvements in clinical outcomes for CR participants, such as changes in LVEF, it is important to acknowledge that these clinical improvements did not translate into significant cost savings or long-term economic benefits within Kazakhstan’s healthcare framework. These findings highlight the need for more substantial investment in CR to make it a truly effective part of post-AMI recovery in Kazakhstan.

This study also explored secondary outcomes related to functional status and workforce participation, revealing that CR users experienced longer average durations of disability and higher rates of persistent disability over two years. At first glance, these results seem counterintuitive, suggesting worse social recovery among CR users. However, these trends likely reflect selection bias, where patients with more severe clinical presentations were preferentially referred to CR.

Indeed, prior studies have consistently shown that CR participants tend to have worse baseline functional statuses but achieve better long-term outcomes [29]. Moreover, the psychosocial and vocational benefits of CR, including structured support, psychological counseling, and return-to-work planning, are well-documented and tend to manifest beyond short-term observation periods [7,9].

For example, a Danish cohort study by Oerkild et al. found that CR participants had a significantly higher likelihood of returning to work at one year compared with non-participants despite similar early disability patterns [30]. In this context, the inclusion of long-term follow-up data (e.g., 3- to 5-year outcomes) in future Kazakh studies would be crucial to fully capture CR’s social return on investment.

Despite compelling clinical and economic arguments, CR utilization remains suboptimal. Only 40.9% of eligible patients in this cohort received CR during the study period, consistent with global participation estimates but indicative of persistent barriers in the Kazakh context. Prior national studies have highlighted issues such as limited program availability outside major urban centers, workforce shortages, and gaps in physician referral [9].

Babayeva et al., reviewing the state of CR in Kazakhstan, emphasized the need for unified clinical guidelines, standardized rehabilitation protocols, and integration of CR into discharge planning processes to boost participation [10]. Similarly, Pesah et al. argue that the success of CR scale-up in middle-income settings requires not only financial support through insurance but also organizational reforms to enhance program accessibility and quality assurance [12]. In addition to these supply-side and referral-side barriers, demographic aging creates a demand-side constraint that can further depress uptake and dilute program impact in the Kazakh context. Age-related declines in cognition, physical capacity, and psychosocial resilience may reduce acceptance of referral and adherence to CR schedules, prolong recovery, and shift resource use toward more intensive medical and social support. Evidence from psychogeriatric research links these vulnerabilities to higher healthcare utilization and expenditures, underscoring the need for age-adapted multidisciplinary CR models to avoid escalating costs and erosion of effectiveness [31].

Our findings lend further urgency to these reforms. In our cohort, participation in phase II cardiac rehabilitation was associated with a modest improvement in left ventricular ejection fraction; however, this change did not translate into measurable reductions in long-term treatment costs or hospital readmissions. Moreover, disability rates were unexpectedly higher among rehabilitation participants compared with non-participants. These findings suggest that while the program may confer certain functional benefits, its overall clinical and economic impact in its current form is limited, underscoring the need for program refinement and further evaluation within the national healthcare context.

The inclusion of CR within Kazakhstan’s compulsory medical insurance system represents a progressive policy shift. However, our findings suggest that insurance coverage alone is insufficient to drive optimal utilization and outcomes. Active measures must accompany financial access to ensure that services are delivered equitably, efficiently, and at a sufficient scale.

Taylor et al. recommend that national insurance schemes prioritize CR by adopting bundled payment models, incentivizing physician referrals, and integrating CR indicators into hospital performance evaluations [32]. Furthermore, expanding tele-rehabilitation and home-based models, especially relevant in Kazakhstan’s rural regions, can increase reach without escalating costs [23,33].

In our study, although CR incurred marginally higher initial expenses, the convergence of cumulative costs over time highlights the potential of insurance mechanisms to absorb short-term spending in exchange for long-term savings. The alignment of financial incentives across providers and payers is therefore critical to the sustainability of CR programs.

### Study Strengths and Limitations

The present cohort study represents the first large-scale analysis of the clinical effectiveness and economic impact of phase II CR within Kazakhstan’s compulsory medical insurance framework. By analyzing a five-year retrospective dataset of 2672 patients with AMI treated at the Almaty City Cardiology Center, this research provides critical evidence supporting the integration and expansion of structured CR services in middle-income health systems. Our findings demonstrate that participation in phase II CR is associated with significant improvements in LVEF and overall cardiac function without incurring higher cumulative treatment costs over a two-year follow-up. These findings are consistent with the international literature, reinforcing that CR is not only clinically beneficial but also economically viable in diverse healthcare contexts [3,4,7,8,11,17,20].

This study benefits from a robust sample size (n = 2672), multi-year follow-up, and comprehensive integration of both clinical and cost data. The retrospective cohort design enabled the analysis of real-world outcomes, enhancing the external validity. Nonetheless, several limitations merit consideration. First, the observational nature of this study precludes definitive causal inference. While statistical adjustment for baseline differences was performed, residual confounding cannot be excluded. Second, data on actual CR adherence (e.g., session attendance, exercise intensity) were unavailable, limiting the ability to assess dose–response relationships. Third, long-term functional and quality-of-life outcomes beyond two years were not captured. Another limitation of our study is the absence of reliable cause-of-death data in the available administrative records. In Kazakhstan, death certification, particularly outside tertiary care centers, often lacks standardized diagnostic coding, making it difficult to differentiate cardiac from non-cardiac causes. As a result, our mortality analyses were restricted to all-cause mortality, which may have obscured disease-specific outcome patterns.

Future studies should employ prospective designs, incorporate patient-reported outcomes, and explore implementation science approaches to optimize CR delivery in Kazakhstan. Additionally, cost-effectiveness modeling using QALYs would provide a more granular understanding of value for money.

## 5. Conclusions

In conclusion, this study reveals that while phase II CR may offer some clinical benefits, the current state of government funding in Kazakhstan is insufficient to allow for these benefits to be realized at a large scale. Our results suggest that CR does not lead to substantial reductions in long-term treatment costs and the financial support allocated for rehabilitation programs is inadequate to produce meaningful outcomes for patients. Given these limitations, it is clear that the funds allocated for CR must be significantly increased to ensure its effectiveness and sustainability. Without such financial improvements, the expansion of CR services will remain hindered, limiting their potential to positively impact the cardiovascular health of the population.

## Figures and Tables

**Table 1:** Baseline socio-demographic and clinical characteristics of patients with acute myocardial infarction in relation to cardiac rehabilitation participation.

		Total	CR Users	Non-CR Users	*p* Value		
Socio-demographic	Age (mean ± SD)	53.8 ± 6.6	53.87 ± 6.6	53.79 ± 6.5	0.766		
	25–34	1.19%					
	35–44	8.57%					
	45–54	35.18%					
	≥55	55.06%					
					**Chi-Square Test**		
					**χ^2^**	**df**	***p* Value**
	Male	90.2%	89%	91.1%	3.124	1	0.077
	Female	9.8%	11%	8.9%			
Education	Incomplete secondary education	3%	2.8%	3.0%	1.298	4	0.862
	Incomplete university education	2%	1.7%	2.2%			
	School education	37%	36.4%	37.4%			
	Secondary specialized education	35.1%	35.5%	34.8%			
	University education	22.9%	23.4%	22.5%			
Residency status	Urban	81.1%	82.7%	80%	3.054	1	0.081
	Rural	18.9%	17.3%	20%			
Living status	Currently alive	87.9%	86.6%	88.8%	2.854	1	0.091
	Currently deceased	12.1%	13.4%	11.2%			
Current disability	Yes	12.3%	14.4%	10.9%	7.290	1	0.007 **
	No	87.7%	85.6%	89.1%			
Remaining on disability for 2 years of observation after MI	Yes	39.7%	43.1%	37.3	9.244	1	0.002 **
	No	60.3%	56.9%	62.7%			
	Mean of disability (mean month ± SD)	8.8 ± 11.2	9.68 ± 11.4	8.21 ± 11			0.001 **
MI occurrence at first hospitalization	Primary MI	78.6%	78.9%	78.4%	0.104	1	0.747
	Recurrent MI	21.4%	21.1%	21.6%			
Type of MI	ST elevation MI (STEMI)	56.1%	58.5%	54.5%	4.244	1	0.039 *
	Non-STEMI	43.9%	41.5%	45.5%			
Treatment received at first hospitalization	Drug treatment	23.1%	21.3%	24.2%	7.194	2	0.027 *
	PCI	60.7%	60.3%	61.0%			
	CABG	16.2%	18.3%	14.8%			

Table Abbreviations: CR, cardiac rehabilitation; SD, standard deviation; BMI, Body Mass Index; LVEF, left ventricular ejection fraction; PCI, percutaneous coronary intervention; CABG, coronary artery bypass grafting. ** *p* < 0.01 and * *p* < 0.05.

**Table 2:** Initial hospitalization metrics and cardiac rehabilitation timelines.

	Yes	No	*p* Value
Cardiac rehabilitation	40.9%	59.1%	<0.001 ***
Initial treatment cost (USD, mean ± SD)	1988.80 ± 1290.1	1879.36 ± 1104.8	0.019 ** 0.019 **
Initial treatment cost (KZT, mean ± SD)	1,046,736.86 ± 679,003.5	989,139.52 ± 581,503.7	
Time taken to complete rehabilitation (months ± SD)	1.89 (±0.49)		

Table Abbreviations: CR, cardiac rehabilitation; SD, standard deviation; USD, United States dollars; KZT: tenge, the currency of Kazakhstan. *** *p* < 0.001 and ** *p* < 0.01.

**Table 3:** Changes in left ventricular ejection fraction (LVEF) among CR and non-CR patients.

	CR Users	Non-CR Users	*p* Value (Independent-Samples *t*-Test)			
Initial LVEF (mean ± SD)	51.75 ± 10.8	47.77 ± 13	<0.001 ***			
Second LVEF (mean ± SD)	53.71 ± 9.2	48.98 ± 10.4	<0.001 ***			
	**Univariate General Linear Model**					
	**Type III SS**	**df**	**MS**	**F**	**Sig.**	**Partial Eta Squared**
Rehabilitation	4173.987	1	4173.987	74.019	<0.001	0.027 *
Initial LVEF (covariate)	117,133.398	1	117,133.398	2077.163	<0.001	0.438
Error	150,507.685	2669	56.391			

Table Abbreviations: CR, cardiac rehabilitation; LVEF, left ventricular ejection fraction; SS, sum of squares; df, degrees of freedom; MS, mean square; F, F-statistic; Sig., significance (*p* value). *** *p* < 0.001, and * *p* < 0.05. R^2^ = 0.467.

**Table 4:** Comparison of clinical outcomes and treatment costs for repeat cardiovascular events within two years post-AMI.

			CR Users	Non-CR Users	χ^2^	df	*p* Value
Repeat MI in same year	Frequency	Yes	43.7%	56.3%	0.424	1	0.515
		No	40.7%	59.3%			
	Treatment received for repeat MI in the same year	Drug treatment	50.0%	50.0%	8.496	7	0.291
		PCI	28.6%	71.4%			
		CABG	49.3%	50.7%			
	Bed days (mean ± SD)		9.44 ± 3.5	9.33 ± 3.6			0.865
	Treatment payment (USD, mean ± SD)		3175.54 ± 1455.5	2955.51 ± 1365.6			0.385
	Treatment payment (KZT, mean ± SD)		1,671,337.78 ± 766,081.1	1,555,535.06 ± 718,787.5			0.385
Repeat MI in the next 2 years after primary hospitalization	Frequency	Yes	39.7%	60.3%	0.074	1	0.786
		No	40.9%	59.1%			
	Treatment received for repeat MI in the same year	Drug treatment	25.0%	75.0%	9.372	6	0.154
		PCI	43.9%	56.1%			
		CABG	100.0%	0.0%			
	Bed days (mean ± SD)		10.14 ± 5	9.01 ± 3.1			0.129
	Treatment payment (USD, mean ± SD)		2169.45 ± 956.3	1745.67 ± 869.3			0.017 *
	Treatment payment (KZT, mean ± SD)		1,141,817.16 ± 503.360.7	918.775.78 ± 457.578.4			0.017 *
Emergency hospitalization for unstable angina during the same year	Frequency	Yes	42.2%	57.8%	0.138	1	0.711
		No	40.8%	59.2%			
	Treatment received for repeat MI in the same year	Drug treatment	38.4%	61.6%	5.062	7	0.652
		PCI	42.0%	58.0%			
		CABG	53.3%	46.7%			
	Bed days (mean ± SD)		8.85 ± 4.1	8.89 ± 4.3			0.943
	Treatment payment (USD, mean ± SD)		1806.79 ± 1329.3	1617.61 ± 1249.8			0.327
	Treatment payment (KZT, mean ± SD)		950.943.34 ± 699.668.8	851.374.24 ± 657.824.2			0.327
Emergency hospitalization for unstable angina in the next 2 years after primary MI	Frequency	Yes	37.6%	62.4%	1.228	1	0.268
		No	41.2%	58.8%			
	Treatment received for repeat MI in the same year	Drug treatment	38.8%	61.2%	6.495	7	0.483
		PCI	34.4%	65.6%			
		CABG	50.0%	50.0%			
	Bed days (mean ± SD)		9.75 ± 5.5	9.14 ± 5.1			0.337
	Treatment payment (USD, mean ± SD)		1443.40 ± 1090.2	1556.78 ± 1053.2			0.556
	Treatment payment (KZT, mean ± SD)		759.686.65 ± 573.816.6	819.359.33 ± 554.334.9			0.560

Table Abbreviations: CR, cardiac rehabilitation; χ^2^, chi-square statistic; df, degrees of freedom; *p*, *p* value; MI, myocardial infarction; PCI, percutaneous coronary intervention; CABG, coronary artery bypass grafting; USD, United States dollars; KZT: Kazakhstani tenge. * *p* < 0.05.

## Data Availability

The datasets used and/or analyzed during the current study are available from the corresponding author on reasonable request.

## Abbreviations

The following abbreviations are used in this manuscript:

CVDs	Cardiovascular diseases
CR	Cardiac rehabilitation
AMI	Acute myocardial infarction
LVEF	Left ventricular ejection fraction
ICD-10	International Classification of Diseases, Tenth Revision
PCI	Percutaneous coronary intervention
CABG	Coronary artery bypass grafting
